# Supplementary material for: Protocol for a theory-based, mixed methods evaluation of Cynnau|Ignite: an active learning programme to foster positive research culture through leadership development at Cardiff University
Source: PLoS One. 2025 Feb 27;20(2):e0319020. doi: 10.1371/journal.pone.0319020 (PMC11867394; doi:10.1371/journal.pone.0319020)
Supplement: S1 File — (DOCX) [file pone.0319020.s001.docx]

Cynnau | Ignite

Cynnwys | Contents

[Deilliannau Dysgu Cynnau | Ignite Learning Outcomes 1](#_Toc542952592)

[Modiwl 1: Diwylliant Ymchwil a Arweinir gan Werthoedd | Module 1: Values-led Research Culture 2](#_Toc1510694404)

[Modiwl 2: Ymchwil Gyfrifol | Module 2: Responsible Research 3](#_Toc1816971153)

[Modiwl 3: Creu Diwylliannau Ymchwil Cynhwysol | Module 3: Creating Inclusive Research Cultures 4](#_Toc1569516858)

[Modiwl 4: Dylanwadu ar Newid | Module 4: Influencing Change 5](#_Toc142257760)

[Modiwl 5: Rhoi Grym i Ymchwilwyr | Module 5: Empowering Researchers 6](#_Toc1600505270)

[Modiwl 6: O Oroesi i Ffynnu - Gyrfaoedd | Module 6: From Surviving to Thriving - Careers 7](#_Toc920256828)

[Modiwl 7: Cysyniadu Ymchwil fel Budd Cyhoeddus | Module 7: Conceptualising Research as a Public Good 8](#_Toc1993182023)

# Deilliannau Dysgu Cynnau | Ignite Learning Outcomes*

| 1. Deall diwylliannau ymchwil yn well ym Mhrifysgol Caerdydd ac yn y sector addysg uwch ehangach. 2. Mynegi’r gwerthoedd a'r ymddygiadau craidd sy'n cyfrannu at amgylchedd ymchwil cadarnhaol ar draws disgyblaethau a llwybrau gyrfa. 3. Cymhwyso arferion arweinyddiaeth, sgiliau newydd, a ffyrdd o weithio i ymgorffori diwylliant ymchwil cadarnhaol. 4. Datblygu rhwydweithiau proffesiynol gyda chydweithwyr o bob rhan o'r Brifysgol i ymgysylltu â materion sy'n ymwneud â diwylliant ymchwil a chyd-greu cynigion arloesol ar gyfer newid. 5. Cynhyrchu syniadau newydd ar gyfer prosiectau ymchwil a/neu ddatblygu sy'n ymgorffori ac sy’n dangos egwyddorion diwylliant ymchwil cadarnhaol yn llawn. | 1. Better understand research culture(s) at Cardiff University and in the wider higher education sector. 2. Articulate the core values and behaviours which contribute to a positive research environment across disciplines and career pathways. 3. Apply leadership practices, new skills, and ways of working to embody a positive research culture. 4. Develop professional networks with colleagues from across the University to engage with issues relating to research culture and co-create innovative proposals for change. 5. Generate new ideas for research and/or development projects which fully incorporate and illustrate the principles of a positive research culture. |
| --- | --- |

*Detailed learning outcomes are iterative and will remain in-design throughout programme

# Modiwl 1: Diwylliant Ymchwil a Arweinir gan

# Werthoedd | Module 1: Values-led Research Culture

**Arweinydd | Leader:** Karin Wahl-Jorgensen

# Modiwl 1: Diwylliant Ymchwil a Arweinir gan

# Werthoedd | Module 1: Values-led Research Culture

**Arweinydd | Leader:** Karin Wahl-Jorgensen

**Disgrifiad Modiwl | Module Description:**

| Mae’r modiwl hwn yn cyflwyno’r cysyniad o ddiwylliant ymchwil ac, yn benodol, y ffactorau sy’n cyfrannu at ddiwylliant cadarnhaol. Byddwn yn trafod sut y gall diwylliant ymchwil effeithio ar unigolion a grwpiau yn ogystal â chyfraniad unigolion at ei gynaliadwyedd a'i ddatblygiad. Byddwn hefyd yn cyflwyno cysyniadau allweddol ym maes arweinyddiaeth a sut y gellir eu cymhwyso at ymchwil. | This module introduces the concept of research culture and, specifically, the factors which contribute to a positive culture. We will discuss how research culture can affect individuals and groups as well the contribution of individuals to its sustainability and development. We will also introduce key concepts in leadership and how they can be applied to research. |
| --- | --- |

**Deilliannau Dysgu | Learning Outcomes:**

| 1. Trafod diwylliannau ymchwil sy'n bodoli yn y Brifysgol. 2. Adnabod ymddygiadau, gwerthoedd ac agweddau cadarnhaol sy'n effeithio ar ddiwylliant ymchwil. 3. Asesu effaith diwylliant ymchwil ar unigolion a grwpiau. 4. Gwerthuso sut y gall unigolion gyfrannu at wella diwylliant ymchwil cadarnhaol a’i gynnal. 5. Dehongli damcaniaethau arweinyddiaeth gan ystyried rôl yr unigolyn. | 1. Discuss research cultures which exist in the University. 2. Recognise positive behaviours, values, and attitudes that affect research culture. 3. Assess the impact of research culture on individuals and groups. 4. Evaluate how individuals can contribute to improving and sustaining a positive research culture. 5. Interpret theories of leadership considering the role of the individual. |
| --- | --- |

#

# Modiwl 2: Ymchwil Gyfrifol | Module 2: Responsible Research

**Arweinydd | Leader:** Roger Whitaker

**Disgrifiad Modiwl | Module Description:**

| Bydd y modiwl hwn yn galluogi cyfranogwyr i ymgorffori egwyddorion ymchwil cyfrifol yn eu gwaith a hyrwyddo’r egwyddorion hyn yn y gymuned ymchwil ehangach. Byddwn yn trafod gonestrwydd a thryloywder yn y broses ymchwil ac yn ystyried rôl cynwysoldeb wrth lunio ymchwil. Mae ail ran y modiwl yn cyflwyno tueddiadau cyfredol mewn arferion ymchwil, megis Deallusrwydd Artiffisial, ac asesiad cyfrifol ymchwil. | This module will enable participants to incorporate the principles of responsible research in their work and advocate for these principles in the wider research community. We will discuss openness and transparency in the research process and consider the role of inclusivity in research design. The second part of the module presents current trends in research practices, such as Artificial Intelligence, and responsible research assessment. |
| --- | --- |

**Deilliannau Dysgu | Learning Outcomes:**

| 1. Crynhoi prif ystyriaethau ymchwil foesegol a sut y gellir eu cymhwyso at ein prosiectau ein hunain. 2. Diffinio'r ffactorau sy'n cyfrannu at arferion ymchwil agored. 3. Trafod sut mae tueddiadau cyfredol, megis twf DA, yn debygol o effeithio ar yr agenda ymchwil. 4. Gwerthuso'r gwerthoedd sy'n sail i asesiad ymchwil cyfrifol. | 1. Summarise the main considerations of ethical research and how they can be applied to our own projects. 2. Define the factors which contribute to open research practices. 3. Discuss how current trends, such as the growth of AI, are likely to impact the research agenda. 4. Evaluate the values which underpin responsible research assessment. |
| --- | --- |

# Modiwl 3: Creu Diwylliannau Ymchwil Cynhwysol | Module 3: Creating Inclusive Research Cultures

**Arweinydd | Leader:** Kerry Hood

**Disgrifiad Modiwl | Module Description:**

| Mae’r modiwl hwn yn gofyn sut mae creu diwylliannau ymchwil cynhwysol. Gofynnir i gyfranogwyr ystyried sut mae rhwystrau yn gallu creu anfantais i gydweithwyr eraill ac a oes modd sicrhau ecwiti i bawb. Clywir am effaith microymosodiadau ac ymddygiadau gwahaniaethol eraill ar gydweithwyr cyn troi at sut y gellir sicrhau ein bod yn creu rhwydweithiau cefnogol yn ogystal â dangos cynghreiriaeth weladwy. | This module asks how we can create inclusive research cultures. Participants will be asked to consider how barriers can create a disadvantage for other colleagues and whether it is possible to ensure equity for all. We will hear about the impact of microaggressions and other discriminatory behaviours on colleagues before turning to how we can ensure that we create supportive networks as well as showing visible allyship. |
| --- | --- |

**Deilliannau Dysgu | Learning Outcomes:**

| 1. Trafod rhwystrau strwythurol a systemig i ecwiti ymhlith staff ym maes Addysg Uwch. 2. Diffinio microymosodiadau ac adnabod ymddygiadau gwahaniaethol. 3. Gwerthuso rôl rhwydweithiau cefnogol mewn cymunedau ymchwil. 4. Cydnabod y ffyrdd y gellir dangos cynghreiriaeth ac arweinyddiaeth weladwy yn y gymuned ymchwil. | 1. Discuss structural and systemic barriers to equity among staff in Higher Education. 2. Define microaggressions and identify discriminatory behaviours. 3. Evaluate the role of supportive networks in research communities. 4. Recognise the ways in which visible allyship and leadership can be shown in the research community. |
| --- | --- |

# Modiwl 4: Dylanwadu ar Newid | Module 4: Influencing Change

**Arweinydd | Leader:** 64 Million Artists External Provider

**Disgrifiad Modiwl | Module Description:**

|  |  |
| --- | --- |

**Deilliannau Dysgu | Learning Outcomes:**

|  |  |
| --- | --- |

**Cynnwys y Modiwl | Module Content:**

|  |  |
| --- | --- |

# Modiwl 5: Rhoi Grym i Ymchwilwyr | Module 5: Empowering Researchers

**Arweinydd | Leader:** Jon Morris

**Disgrifiad Modiwl | Module Description:**

| Mae'r modiwl hwn yn canolbwyntio ar ddatblygiad personol a bydd yn rhoi cyfle i gyfranogwyr adfyfyrio ar eu gyrfaoedd a'u bywydau gwaith eu hunain hyd yn hyn yn ogystal ag ennill sgiliau a syniadau ar gyfer ffyrdd newydd o weithio. Byddwn yn dechrau gyda thrafodaeth onest am yr heriau sy'n wynebu ymchwilwyr, yn enwedig y rheini ym mlynyddoedd cynnar eu gyrfaoedd. Byddwn yn gweithio gyda'n gilydd i ddatblygu pecyn cymorth o adnoddau i gefnogi datblygiad gyrfa a lles a chlywed gan eraill am eu profiadau. | This module focuses on personal development and will allow participants the opportunity to reflect on their own careers and working lives so far as well as gain skills and ideas for new ways of working. We will begin with an honest discussion of the challenges facing researchers, especially those in the early years of their careers. We will work together to develop a toolkit of resources to support career development and wellbeing and hear from others about their experiences. |
| --- | --- |

**Deilliannau Dysgu | Learning Outcomes:**

| 1. Nodi'r materion cyffredin sy'n effeithio ar ymchwilwyr. 2. Cynhyrchu syniadau ar sut y gallant reoli eu prosiectau ymchwil a'u proffil yn effeithiol. 3. Dangos sut i reoli a goresgyn rhwystrau. 4. Nodi cyfleoedd ar gyfer cydweithio a meithrin colegoldeb. | 1. Identify the common issues that affect researchers. 2. Generate ideas on how they can effectively manage their research projects and profile. 3. Demonstrate how to manage and overcome setbacks. 4. Identify opportunities for collaboration and cultivating collegiality. |
| --- | --- |

# Modiwl 6: O Oroesi i Ffynnu - Gyrfaoedd | Module 6: From Surviving to Thriving - Careers

**Arweinydd | Leader:** Carrie Lear

**Disgrifiad Modiwl | Module Description:**

| Yn y modiwl hwn, bydd cyfranogwyr yn trafod eu canfyddiadau eu hunain o yrfaoedd yn y gymuned ymchwil ac yn dysgu sut i osod nodau gyrfa hirdymor. Byddant yn gallu pwyso a mesur y llwybrau gyrfa sydd ar gael ac alinio hynny â'r sgiliau sydd eu hangen ar gyfer cynllunio gyrfa. Bydd cyfranogwyr yn dysgu gan arweinwyr sydd wedi dilyn llwybrau gyrfa amrywiol. Bydd y modiwl hwn hefyd yn datblygu sgiliau cyfranogwyr wrth lywio sgyrsiau gyrfa a defnyddio rhwydweithiau. | In this module, participants will discuss their own perceptions of careers in the research community and learn how to set long-term career goals. They will be able to assess available career paths and align that with the skills needed for career planning. Participants will learn from leaders who have followed diverse career paths. This module will also develop participant’s skills in navigating career conversations and leveraging networks. |
| --- | --- |

**Deilliannau Dysgu | Learning Outcomes:**

| 1. Gwerthuso'r cyfleoedd datblygu perthnasol sydd ar gael i ymchwilwyr. 2. Defnyddio cymorth rhwydweithiau mewnol ac allanol yn y sector addysg uwch a thu hwnt. 3. Mynegi eu nodau mewn sgyrsiau gyrfa cadarnhaol. 4. Dehongli sgiliau angenrheidiol o fewn llwybrau gyrfa amrywiol a’u halinio â’u nodau datblygu unigol. | 1. Evaluate the relevant development opportunities available for researchers. 2. Leverage internal and external networks in the higher education sector and beyond. 3. To articulate their goals in positive career conversations. 4. To interpret necessary skills within diverse career paths and align them with their individual development goals. |
| --- | --- |

# Modiwl 7: Cysyniadu Ymchwil fel Budd Cyhoeddus | Module 7: Conceptualising Research as a Public Good

**Arweinydd | Leader:** Claire Gorrara

**Disgrifiad Modiwl | Module Description:**

| Ystyried hunaniaeth ymchwilwyr a’u perthynas â’r cyhoedd yw nodau’r modiwl hwn. Yn gyntaf, byddwn yn ystyried rolau academyddion yn ym mywyd cyhoeddus gan ganolbwyntio ar y cysyniadau o eiriolaeth ac actifiaeth. Yn ail, eir ati i drafod sut y gellir creu perthnasau â defnyddwyr ymchwil a chymunedau i greu traweffaith. Mae’r modiwl yn cloi drwy ofyn i gyfranogwyr ystyried eu hunaniaeth fel ymchwilwyr ac i ba raddau y gellid ymgorffori agweddau ar y hyn a drafodwyd yn eu bywydau. | The aims of this module are to consider the identity of researchers and their relationship with the public. Firstly, we will consider the roles of academics in public life focusing on the concepts of advocacy and activism. Secondly, we will discuss how relationships can be created with research users and communities to create impact. The module concludes by asking participants to consider their identity as researchers and the extent to which aspects of what was discussed could be incorporated into their lives. |
| --- | --- |

**Deilliannau Dysgu | Learning Outcomes:**

| 1. Trafod rôl actifiaeth ymhlith ymchwilwyr a gwahaniaethu rhwng eiriolaeth a gweithrediaeth. 2. Disgrifio pwysigrwydd gwerth cyhoeddus ar lefel sefydliadol ac unigol. 3. Cydnabod y ffyrdd amrywiol o ymgysylltu â defnyddwyr ymchwil, galluogwyr, a chymunedau ehangach i annog cyd-greu a thraweffaith. 4. Gwerthuso eu cyfraniad i'r gymuned ehangach a bywyd cyhoeddus. | - 1. To discuss the role of activism among researchers and differentiate between advocacy and activism.   2. To describe the importance of public value at institutional and individual levels.   3. To recognise the diverse ways of engaging with research users, enablers, and wider communities to encourage co-creation and impact.   4. To evaluate their contribution to the wider community and public life. |
| --- | --- |
