# Supplementary material for: Protocol for a theory-based, mixed methods evaluation of Cynnau|Ignite: an active learning programme to foster positive research culture through leadership development at Cardiff University
Source: PLoS One. 2025 Feb 27;20(2):e0319020. doi: 10.1371/journal.pone.0319020 (PMC11867394; doi:10.1371/journal.pone.0319020)
Supplement: S2 File — (DOCX) [file pone.0319020.s002.docx]

## S2. Cynnau|Ignite Evaluation Materials Table

| **Data Collection Period** | **Materials and Source** |
| --- | --- |
| Baseline (T1):  Background | Participants’ background and demographics, including:   - **Employment Contract** *(open-ended / open-ended with relevant factor / temporary or fixed-term / prefer not to say)* - **Employment Department or School** *(within Cardiff University)* - **Age** *(I am… / prefer not to say)* - **Disability or Long-Term Health Condition** *(yes / no / prefer not to say)* - **Ethnicity** *(Asian, Asian English, Scottish, Welsh, or Northern Irish / Black, African, Carribean, Black English, Scottish, Welsh, or Northern Irish / Mixed, multiple ethnicity / White, White English, Scottish, Welsh, or Northern Irish, any other white* *background / other ethnic group - please describe / prefer not to say)* - **Gender** *(man / woman / non-binary / prefer not to say)* - **Identify as Trans** *(yes / no / prefer not to say)* - **Sexual Orientation** *(asexual spectrum / bisexual or bi+ / gay man / gay woman or lesbian / heterosexual / queer / prefer to self-describe / prefer not to say)* - **Pregnancy** *(yes / no / prefer not to say)* - **Recent Period of Leave** *(yes / no / prefer not to say)* - **Caring Responsibilities** *(yes / no / prefer not to say)* - **Religion or Belief** *(no religion / Buddhist / Christian / Hindu / Jewish / Muslim / Sikh / any other religion – please describe / prefer not to say)* - **Welsh Ability** *(yes, happy to use in work environment / yes, not happy to use in work environment / yes, in a limited way / no, only a few words / no, not at all / other / prefer not to say)*   Background and demographics questions were developed in accordance with Cardiff University’s Research Culture Survey [1]. |
| Baseline and Follow-Up (T1,2,3):  Research Culture Practices | Participants’ attitudes, subjective norms, perceived behavioural control, and actual behaviour relative to Positive Research Culture (PRC) practices, organised by six dimensions of the UKRI PRC Framework. These are:   - **Open Research** - **Research integrity** - **Communicating Research** - **Inclusive Work Environments** - **Building Collegiality** - **Realising Impact**   For each dimension, participants are given the UKRI definition of such practices, and asked whether they carried out these practices in the last six months, and to list all instances (if applicable). This section was designed specifically for the evaluation, and based on the UKRI PRC Framework [2]. Dimensions listed here were chosen because of their relevance to Cynnau\|Ignite’s modules and learning outcomes (S1).  Marked on 7pt Likert from *Strongly Disagree (1)* to *Strongly Agree (7)* */ Not Applicable (8)*, participants are then asked whether these practices are:   - **Useful / Valuable** (A) - **Enjoyable** (A) - **Common among peers** (N) - **Expected by managers** (N) - **Expected by the university** (N) - **Dependant only on them** (PBC) - **Achievable if desired** (PBC)   Finally, participants are asked whether they intend to carry out these practices in the next six months, marked on 7pt Likert from *Strongly Disagree (1)* to *Strongly Agree (7) / Not Applicable (8)*, and to justify these intentions (open text).  Specific questions were developed in accordance with the Theory of Planned Behaviour (TPB) survey guidelines [3]. In-line with TPB, we sought to measure participants’ attitudes (items marked ‘A’), social norms (N), and perceived behavioural control (PBC) which are thought to contribute to the formation of behavioural intentions [4]. The sum of these responses will produce a single score in each category (Attitudes, Norms, and Perceived Behavioural Control) within each UKRI dimension [2]. |
| Baseline and Follow-Up Surveys:  Experiences at Work | Participants’ experiences at work and job satisfaction are marked on 5pt Likert from *Never (1)* *to Very Often (5) / Not Applicable (6)*. Questions include:   - **Feeling ignore or excluded** - **Heavy workloads** - **Time pressure** - **Job security** - **Job satisfaction**   These questions were sourced from Matthews et al. [5] and chosen to reflect the common issues cited in Cardiff University’s Research Culture Survey [1] |
| Baseline, Follow-Up 1 and Follow-Up 2 Surveys: | Based on a broad definition of Community of Practice from Harvard Business Review [6], participants are asked whether they:   - **Consider themselves part of a PRC-related Community of Practice?** - **Feel supported by their CoP (if applicable)?**   Responses were marked on 7pt Likert from *Not at All (1)* to *A Great Deal (7) / Not Applicable (8)*. These items can stand alone, or be averaged to produce a single CoP score. |
| Baseline, Follow-Up 1 and Follow-Up 2 Surveys | Participants are asked about research skills targeted by Cynnau\|Ignite, specifically:   - **Leadership** (L) - **Career ownership** (EI) - **Creativity** (EI) - **Ability to speak out without repercussions** (EI) - **Professional development** (P/CD) - **Autonomy and responsibility** (EI) - **Ambition** (EI) - **Self-reflection** (EI) - **Risk-taking** (RA)   Responses are marked on a 7pt Likert from *Strongly Disagree (1)* to *Strongly Agree (7) / Not Applicable (8)*. Skills represented here were considered those most likely to be influenced by Cynnau\|Ignite according to its module learning outcomes (S1), which focus heavily on Empowering Individuals. These items were mapped to the UKRI PRC Framework dimensions [2] as follows:   - **Recognition and Assessment** (RA) - **Embedding Professional and Career Development** (P/CD) - **Empowering Individuals** (EI)   Items marked RA or PC/D will represent participant scores for Recognition and Assessment and Embedding Professional and Career Development respectively. The average of EI items will represent a score for Empowering Individuals. As self-leadership is heavily emphasised throughout training modules, participants are asked whether they see themselves as a leader. This standalone item (L) will represent a Leadership score. |
| Follow-Up 1 and Follow-Up 2 | Attendance of other PRC initiatives was assessed by asking participants to:   - Indicate the number of other PRC initiatives attended while taking part in Cynnau\|Ignite - If applicable, describe each of these initiatives   These items were created for the purpose of this survey, to mitigate the risk of other PRC initiatives (which have the potential to become more common in Higher Education Institutions) influencing Cynnau\|Ignite outcomes. |
| Stream Evaluation | Stream evaluation forms, modified from Rowbottom et al. [7] for use in the Cynnau\|Ignite project, consist of two matrix tables. The first, marked on a 5pt Likert scale from *Not at All (1)* to *Extreme Amount (5)*, asks whether, during each stream, participants…   - **Benefitted** - **Learned** - **Struggled** - **Became bored**   In the second matrix table, scored on a 5pt Likert scale, from *Strongly Disagree (1)* to *Strongly Agree (5)*, participants must indicate their agreement that:   - **Module length was appropriate** - **Content was sufficient in guiding further Cynnau\|Ignite projects** (if applicable) - **Content helped them understand how to apply their knowledge** - **Content was useful in work/practice** - **They would like to receive similarly designed training** - **They feel ready to use the imparted knowledge** - **The stream was well-organised** - **They are satisfied with the training received**   Finally, participants are asked whether they would recommend *(Yes / No / Unsure)*.  Items from both matrix tables can stand alone, or the sum (with ‘Struggled’ and ‘Become bored’ items reversed) will represent an overall Stream Satisfaction score. |
| Observation | Structured observation sessions utilise a modified version of Lane and Harris’ tool for observation [8], adapted to fit staff training sessions. Each observer is issued a copy of this tool, which contains the following behaviours which are typically related to engagement or disengagement, along with example to look out for:   - **Listening E** *(learner is making eye contact with contributor, smiling, nodding in agreement, leaning forward).* - **Writing** **E** *(learner is taking notes on material, the timing of which relates to the contributor’s presentation or statements. Learner is following along on computer or taking class notes in a word processor).* - **Reading E** *(learner is reading material related to the session. When a question is posed, they flip through their notes).* - **Engaged Learner Interaction E** *(learner discussion relates to class material (verbal/nonverbal behaviour indicates they are listening or explaining lecture content, e.g., using hand gestures or pointing at notes or screen).* - **Engaged with Instructor E** *(learner participates/follows in relevant discussions with instructor).* - **Off-task D** *(learner is unpacking, organizing notes, finding a seat, or packing up and leaving classroom. Learner is off-task (e.g., using their phone, talking to others where discussion does not relate to session material).* - **Not Responding to Contributor D** *(learner is not responsive to contributor. Eyes are closed or not focused. Learner is slouched, sleeping, or unresponsive to contributor’s cues. Learner is observing other Learner(s) and is distracted by an off-task conversation or by another Learner).*   At five ten-minute intervals, observers glance around the room and mark the number of participants (out of the maximum consenting participants) displaying these behaviours. At each timepoint, observers have space to note any other factors which may affect participant engagement. Items marked E will be combined to produce an Engagement score, and D items will form a Disengaged score. Multiple observers will take part in a calibration training session before observation, and scores from all observers will be averaged (with the exception of missed timepoints or outliers). |
| Process Evaluation | Semi-structured interviews will be organised in accordance with, and following the structure of, Table 1. Cynnau\|Ignite evaluation outcomes. Based on realist principles [9,10], this process evaluation has been designed to qualitatively determine the context and population in which Cynnau\|Ignite has potential to succeed. We have considered the programme’s design in relation to its underpinning Theory of Change, as well as barriers and facilitators to implementation and expansion [11,12]. |

## References:

1. Research culture survey report 2023 executive summary - Cardiff University [Internet]. Cardiff University. 2023 [cited 2024 Sep 26]. Available from: <https://www.cardiff.ac.uk/documents/2741029-research-culture-survey-report-2023-executive-summary>
2. UK Research and Innovation. Research financial sustainability: issues paper. www.ukri.org. 2023. Available from:

https://www.ukri.org/publications/research-financial-sustainability-data/research-financial-sustainability-issues-paper/.

1. Ajzen I. Constructing a theory of planned behavior questionnaire. 2006. Available at People. Umass. Edu/Aizen/Pdf/Tpb. Measurement. Pdf.
2. Ajzen I. The theory of planned behavior. Organizational behavior and human decision processes. 1991 Dec 1;50(2):179-211.
3. Matthews RA, Pineault L, Hong YH. Normalizing the use of single-item measures: Validation of the single-item compendium for

organizational psychology. Journal of Business and Psychology. 2022 Aug;37(4):639-73. Wenger EC, Snyder WM.

1. Communities of Practice: The Organizational Frontier [Internet]. Harvard Business Review. 2001. Available from: https://hbr.org/2000/01/communities-of-practice-the-organizational-frontier
2. Rowbottom B, Galhardo A, Donovan E, Gameiro S. Feasibility randomized controlled trial of a self-guided online intervention to promote

psychosocial adjustment to unmet parenthood goals. Human Reproduction. 2022 Oct 1;37(10):2412-25.

1. Lane ES, Harris SE. A new tool for measuring student behavioral engagement in large university classes. Journal of College Science

Teaching. 2015 Jul 1;44(6):83-91.

1. Manzano A. The craft of interviewing in realist evaluation. Evaluation. 2016 Jul;22(3):342-60.
2. Maxwell JA. Collecting qualitative data: A realist approach. The SAGE handbook of qualitative data collection. 2018:19-32.
3. Grant A, Bugge C, Wells M. Designing process evaluations using case study to explore the context of complex interventions evaluated in

trials. Trials. 2020 Dec;21:1-0.

1. Saunders RP, Evans MH, Joshi P. Developing a process-evaluation plan for assessing health promotion program implementation: a how-to

guide. Health promotion practice. 2005 Apr;6(2):134-47.
